# Adenine oligomer directed synthesis of chiral gold nanoparticles

**DOI:** 10.1038/s41467-022-31513-y

**Published:** 2022-07-02

**Authors:** Nam Heon Cho, Young Bi Kim, Yoon Young Lee, Sang Won Im, Ryeong Myeong Kim, Jeong Won Kim, Seok Daniel Namgung, Hye-Eun Lee, Hyeohn Kim, Jeong Hyun Han, Hye Won Chung, Yoon Ho Lee, Jeong Woo Han, Ki Tae Nam

**Affiliations:** 1 Department of Materials Science and Engineering, Seoul National University, Seoul, 08826 Republic of Korea; 2 Department of Chemical Engineering, Pohang University of Science and Technology (POSTECH), Pohang, Gyeongbuk 37673 Republic of Korea; 3 Department of Chemistry, Seoul National University, Seoul, 08826 Republic of Korea

**Keywords:** DNA nanostructures, Nanoparticles

## Abstract

Precise control of morphology and optical response of 3-dimensional chiral nanoparticles remain as a significant challenge. This work demonstrates chiral gold nanoparticle synthesis using single-stranded oligonucleotide as a chiral shape modifier. The homo-oligonucleotide composed of Adenine nucleobase specifically show a distinct chirality development with a dissymmetric factor up to g ~ 0.04 at visible wavelength, whereas other nucleobases show no development of chirality. The synthesized nanoparticle shows a counter-clockwise rotation of generated chiral arms with approximately 200 nm edge length. The molecular dynamics and density functional theory simulations reveal that Adenine shows the highest enantioselective interaction with Au(321)^R/S^ facet in terms of binding orientation and affinity. This is attributed to the formation of sequence-specific intra-strand hydrogen bonding between nucleobases. We also found that different sequence programming of Adenine-and Cytosine-based oligomers result in chiral gold nanoparticles’ morphological and optical change. These results extend our understanding of the biomolecule-directed synthesis of chiral gold nanoparticles to sequence programmable deoxyribonucleic acid and provides a foundation for programmable synthesis of chiral gold nanoparticles.

## Abstract (web summary)

Chiral plasmonic nanoparticles are of great interest in nanotechnology. Here, the authors demonstrate chiral shape guidance by single-stranded oligonucleotides during particle growth based on sequence-specific hydrogen bonding within the strand.

## Introduction

Chirality, the geometrical orientation of matter being non-superimposable to their mirror images, exists in various size scales of the biological system. Its unique optical response with respect to circularly polarized light aroused significant attention to creating artificial chiral nanostructures using plasmonic materials with strong light-matter interactions^1–4^. Artificial plasmonic chiral nanostructures enhance the versatility of electromagnetic fields near the surface, which can be utilized for sensitivity amplification in chirality detection^5–14^ or enantioselective catalytic reaction^15–17^. In chiral sensing, several studies proposed the biosensor based on localized surface plasmon resonance(LSPR) phenomena utilizing shape-engineered chiral plasmonic nanostructures^5–7^. From these studies, changes in the surrounding media, such as refractive index, were detected with higher sensitivity using chiroptical spectroscopic methods. Furthermore, the strong enhancement of near-field optical chirality in the vicinity of chiral plasmonic nanostructures showed amplified sensitivity for chiral discrimination^8–14^. Chiral plasmonic nanostructures also served as a platform for heterogeneous chiral catalysis. With the handedness-dependent interaction of substrate and the nanostructures, chirality was transferred from inorganic materials to prochiral organic substrates, showing broad applicability of chiral plasmonic nanostructures^15–17^.

In this regard, imbuing helicity to plasmonic nanomaterials using chiral biomolecules is widely pursued^18–21^. Recently, long-range assembly of gold nanorods in a helical arrangement using human islet amyloid polypeptides has presented a well-defined nanogap between two edges of the nanorods to enhance its chiroptic response significantly^21^. Alternative to assembly techniques, the introduction of chiral biomolecules during nanocrystal growth generated gold nanoparticles with intrinsic structural chirality^22–26^ The mixed micelles using CTAC and (R or S)-(+)-1,1′-binaphthyl-2,2′-diamine adsorbed on gold nanorods directed the formulation of sharp chiral wrinkles which exhibit strong chiroptic response in NIR region^26^. Furthermore, the chemical synthesis of chiral gold nanoparticles using thiol-containing amino acids and peptides under high-index nanoparticle synthesis conditions achieved well-defined chiral morphologies with strong optical response at visible wavelength^25^. Such methodology of directly transferring molecular chirality to inorganic nanomaterials showed immense potential for chiral nanomaterials synthesis and their diversification, leading to an extended field of applications.

The Deoxyribonucleic acid (DNA) oligomers, composed of 4 types of nucleobases such as Adenine (A), Thymine (T), Cytosine (C), and Guanine (G), are specifically highlighted for their precise nano-structuring capabilities. Three representative characteristics of DNA as a building block for nanomaterials are as follows: (1) facile sequence programmability, (2) specific hybridization properties using complementary sequences, and (3) base-dependent surface interaction on inorganic materials. The facile sequence programmability and highly specific hybridization properties of DNA have been utilized to design chiral assembly structures^27–32^. Yan et al. utilized the oligomer-specific hybridization of DNA to assemble four different nanoparticles in a tetrahedral pyramidal shape with distinct chirality^30^. Designing of attached oligomer sequences on each type of nanoparticles allowed the construction of a chiral assembly structure with four distinct constituent nanoparticles of different sizes or materials. On the contrary, nucleobase-dependent interaction of single-stranded DNA (ssDNA) oligomers with gold metal surfaces has opened up an avenue for synthetic control of nanomaterials. The kinetics and dynamics of surface interaction between oligomers and gold surfaces show a clear base-dependent relationship. Among nucleobases, Adenine is known to show the highest affinity with gold surfaces, followed by Cytosine, Guanine, and Thymine in respective order confirmed through various simulation and experimental methods^33,34^. Such nucleobase-dependent surface interaction induces single-nanoparticle morphology control in Au monometallic and Au-Pd bimetallic system^35–37^. However, up to these dates, nucleobase-dependent surface interaction study has not been understood from the perspective of chirality-inducing effect and chiral morphology controllability.

Herein, we experimentally demonstrated Adenine-specific chirality evolution in chemically synthesized gold nanoparticles. The introduction of Adenine oligomer during nanoparticle synthesis induces chiral morphology evolution with a dissymmetric factor up to 0.04 at the visible wavelength region. On the contrary, all other nucleobases containing deoxyribose sugar molecules with chiral centers showed no chirality-inducing capability. The molecular dynamic (MD) simulations and density functional theory (DFT) calculations were conducted to decipher this Adenine-specific chirality evolution. Simulation results reveal that the favorable formation of the intra-base hydrogen bond in Adenine compared to other bases induces enantioselective interaction and controls the oligomer’s relative orientation with respect to the high-index gold surface. Such base-dependent enantioselectivity has been further extended to sequence modulation of ssDNA oligomers to induce variations of nanoparticle morphology and chiroptic response.

## Results

### Adenine oligomer-specific evolution of chirality in single gold nanoparticle

Aqueous-based two-step growth method has been utilized for ssDNA oligomer-driven chiral nanoparticle synthesis. As the first step, (111) facet-enclosed uniform octahedral nanoparticles with around 40 nm edge length have been synthesized^38^. In the second step, single-stranded DNA (ssDNA) was added to imbue chirality to gold nanoparticles. The typical chiral nanoparticle growth solution contains HAuCl_4_ as the metal ion source, Hexadecyltrimethylammonium bromide (CTAB) as the surfactant, and ascorbic acid as the reducing agent, in which pre-synthesized uniform octahedron nanoparticles evolve into high-index nanoparticles^25^. Achiral high-index-faceted nanoparticle with ({*hkl*}, *h* ≠ *k* ≠ *l* ≠ 0, e.g. (321)) intrinsically contains an equal ratio of chiral surfaces in R and S orientation due to atomic orientation at the kink site. These chiral atomic kink sites provide a platform for enantioselective adsorption between chiral molecules and chiral surfaces^39,40^. Upon addition of chiral inducing molecules such as thiol-containing chiral amino acids and peptides, asymmetric elongation of chiral facet induces overall helical morphologies with fourfold, threefold, and twofold rotational symmetry, named 432 helicoid nanoparticles. In the growth solution, ssDNA oligomers were introduced to induce chirality-dependent surface interaction between ssDNA oligomer and the gold surface, controlling the growth pathway of nanoparticles to exhibit oligomer-dependent nanomorphology and optical properties. For a comprehensive understanding of the role of ssDNA during the ssDNA-induced chirality evolution, oligomer concentration, oligomer length, and oligomer composition were modulated. The change in chirality evolution was understood through Scanning Electron Microscopy (SEM) and Circular Dichroism (CD) spectrometers. The quantification of chirality in synthesized nanoparticles was compared through the dissymmetric factor, *g*-factor, a dimensionless quantity calculated from the measured extinction and CD values. Furthermore, MD simulation and DFT calculations were conducted to understand the specific enantioselective interaction between high-index surface and ssDNA.

**Fig. 1:**
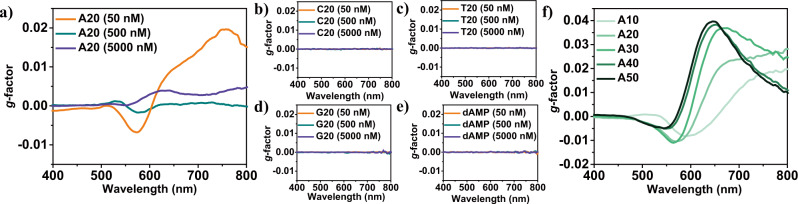
Adenine-specific chirality evolution with sequence length modulation. **a** The chiroptic response of chiral gold nanoparticles synthesized using a 20-mer length Adenine oligomer. concentration The chiroptic response of gold nanoparticle synthesized using 20-mer length (**b**) Cytosine oligomer, (**c**) Thymine oligomer, and (**d**) Guanine oligomer. **e** The chiroptic response of gold nanoparticle synthesized using deoxyadenosine monophosphate monomer. **f** The chiroptic response of gold nanoparticles synthesized with Adenine oligomer sequence length ranging from 10-mer to 50-mer. In the figure legends, A indicate Adenine, C indicate Cytosine, T indicate Thymine, and G indicate Guanine, while numbers indicate the length of the oligomer.

Introduction of Adenine oligomer to growth condition for high-index generation showed distinct chiroptic evolution at visible and NIR wavelength over broad oligomer concentrations, as shown in Fig. 1a. More specifically, Adenine oligomers of 50 nM and 5000 nM showed a chiroptic response between visible wavelength regions of 500 nm to NIR regions of 800 nm. Synthesized chiral gold nanoparticles using 500 nM concentrations of Adenine 20-mer showed reproducible chiroptic response with high nanoparticle stability. As shown in Supplementary Figs. 1 and 2, the representative spectral features of the first negative peak at around 570 nm and a positive peak at around 750 nm have been retained over three repetitive experiments and one month of storage time. Interestingly, this chirality evolution capability of ssDNA oligomers is only observable in Adenine oligomers. Figure 1b–d shows a chiroptic measurement of nanoparticles synthesized using Thymine 20-mer oligomer, Cytosine 20-mer oligomer, and Guanine oligomer. Using each ssDNA oligomer, the synthesized nanoparticles showed no chiroptic response evolution in all tested molecular concentration ranges.

In order to understand the mechanism, chirality evolution has been tested using deoxyadenosine monophosphate monomers. As shown in Fig. 1e, nanoparticles synthesized using deoxyadenosine monophosphate (dAMP) monomers did not show any chirality evolution at all concentration ranges. They only showed the synthesis of trisoctahedral and bipyramid morphologies, as shown in Supplementary Fig. 3. Contrary to the case of dAMP monomers, the chiroptic response of nanoparticles synthesized using Adenine oligomer showed high dependency on the oligomer overall structures. For example, while retaining the final concentration in the growth solution, the length of Adenine oligomer used for the nanoparticle synthesis varied from 10-mer to 50-mer. As shown in Fig. 1f, the chiroptic response showed a gradual decrease, and a blue-shift of negative CD peak was found around 550 nm with the increase of Adenine oligomer length. Furthermore, a positive peak found around 700 nm gradually blue-shifted with an intensity increase up to a g-factor of 0.04 at near 635 nm with an increase of Adenine oligomer length from 10-mer to 50-mer. These results indicate that chirality development is induced by the whole structure of Adenine oligomers and their conformation on the gold surface, which will be further explained by simulation results included in this current work. This chirality evolution mechanism is different from that of our previously reported chiral gold nanoparticles, in which molecular chirality of biomolecules was transferred to inorganic surfaces by enantioselective binding to chiral high-index surfaces^25^.

Relative orientation and surface interaction of Adenine oligomers are closely affected by the surface-bound surfactant, CTAB. The current synthetic system utilizes CTAB molecules as a capping agent and a (100) facet stabilizer, allowing dynamic competition with Adenine oligomers at the organic-inorganic interface to influence nanoparticle morphology control directly. As shown in Supplementary Fig. 4, two-dimensional diagrams of max g-factor at both negative and positive CD peaks have been plotted with respect to changes in CTAB and Adenine oligomer concentration. With the increase in CTAB concentration from 7.5 mM to 37.5 mM, maximum g-factors of both negative (denoted as Peak 1) and positive (denoted as Peak 2) peaks gradually shift towards higher concentration of 50-mer Adenine oligomers from 250 nM to 1000 nM. Furthermore, relative CTAB concentration dictated the dominant sign of CD peak development, where the highest negative peak (peak 1) was observed at 15 mM of CTAB concentration while the highest positive peak (peak 2) was observed at 30 mM CTAB concentration. These results indicate a specific concentration ratio between Adenine oligomer and CTAB molecules is required for the most effective chirality evolution.

**Fig. 2:**
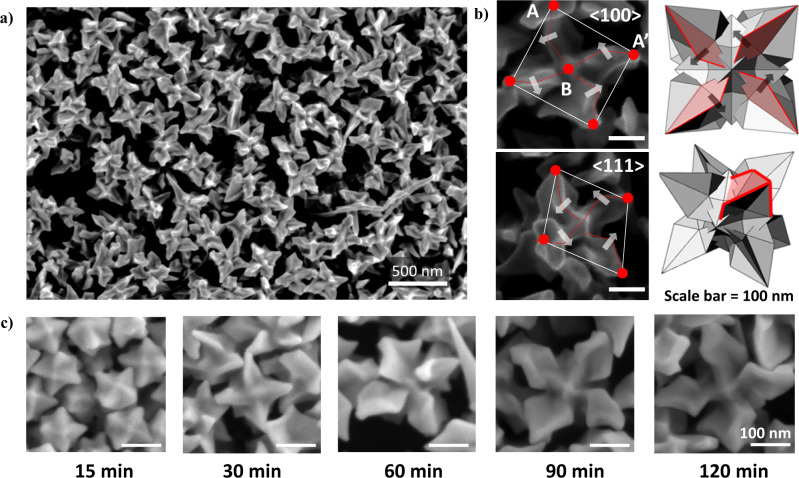
Morphology analysis of Adenine oligomer-induced chiral gold nanoparticles. **a** Low-magnification SEM image of chiral gold nanoparticles synthesized with A50 oligomers. **b** (Left) High-magnification SEM image of chiral gold nanoparticle synthesized with A50 oligomer viewed from <100> and <111> direction. (Right) 3D modeling of chiral gold nanoparticles synthesized from A50 oligomers from <100> and <111> perspective with chiral motif shaded in red. A point denotes the corner point of the nanoparticle and B point denotes the center point of the nanoparticle. The generation of the chiral motif is marked with a red dotted line and the rotational direction is marked with a white arrow. **c** Time-variant morphology SEM images of chiral gold nanoparticles synthesized with A50 oligomers.

The chiroptic response of nanostructures could be closely related to their inherent morphology. Figure 2a shows Adenine-induced chiral gold nanoparticles’ morphology analysis based on low- and high-magnification Scanning Electron Microscopy (SEM) images. Low-magnification images of A50 oligomer-induced chiral gold nanoparticles show uniform morphology with around 220 nm edge length size with each chiral feature protruding outwards in <111> direction. A more detailed structural analysis based on high-magnification images and schematic modeling of synthesized nanoparticle morphology is shown in Fig. 2b. Individual nanoparticle images are viewed and analyzed from <100> and <111> viewpoints to 3-dimensionally visualize the nanoparticle. Viewing the nanoparticle from a <100> perspective, the boundary between a corner point denoted as A and a center protruded point B rotates in a counter-clockwise rotation, composing a chiral feature defined as a chiral arm. There are four chiral arms with the same rotational direction in a counter-clockwise direction, and each chiral arm rotates around the center protruded point B. View from <111> perspective allows a more precise understanding of Adenine-induced chiral gold nanoparticles’ outer boundaries. Each chiral arm is protruded in a <111> direction to show an octopod-like outer boundary with representative chiral features observed from a <100> perspective existing in all six faces of nanoparticles to exhibit unified chirality in 3-dimensional geometry. Furthermore, as shown in Supplementary Fig. 5, change in synthetic conditions strongly affects the chiral nanoparticle morphology. With the increase in CTAB concentrations, while other synthetic conditions have remained constant, an increase in CTAB concentration induces a more profound gap and more acute angle between chiral features, as denoted in the white line. As shown in Supplementary Table 1, a CTAB concentration of 15 mM yields an average of 130.82° (±3.75°) between corner points. With the increase of CTAB concentrations to 22.5 mM and 30 mM, the angle between two corner points decreases to 115.76° (±4.19°) and 101.52° (±7.63°), respectively. This data suggests that relative surface concentrations of CTAB and Adenine oligomers significantly impact the developmental stage of chiral morphology evolution, hence crucially affecting the resulting optical response.

Observing the progress of nanoparticle growth allows insights into mechanisms of chirality development. Previous amino acid and peptide-induced chiral gold nanoparticle synthesis followed hexoctahedron intermediate morphology with a gradual manifestation of chiral features as synthesis time progressed. In 432 helicoid III synthesis using octahedron seed and glutathione molecules, high-index planes and chiral motif generation is followed by more defined chiral feature generation to induce a steep incline of chiroptic response^41^. Adenine-induced chiral gold nanoparticle follows a similar growth pathway and chiroptic response development to 432 helicoid III, as shown in the time-dependent observation of chiral nanoparticle growth in Fig. 2c. In the first 15 min of synthesis, the nanoparticle exhibits high-index formation, which shares a similar intermediate to 432 helicoid III. After 30 min of reaction, the chiral motif of counter-clockwise rotation of boundary starts to show with an increase of edge length to around 120 nm. At 60 min of nanoparticle growth, representative chiral arm generation becomes more defined by increasing nanoparticle size to 190 nm edge length. Further progression of nanoparticle growth to 90 min and 120 min of reaction time finalizes nanoparticle morphology with 220 nm edge length and defined chiral arm formation. Such morphological development can be correlated to the temporal evolution of optical response shown in Supplementary Fig. 6. The chiroptic response starts to evolve between 15 min and 30 min of nanoparticle growth, where chiral tilting of the boundary occurs. Between 30 min and 60 min of reaction time, when vigorous nanoparticle size increases and defined chiral arm generation are observed, the most significant increase in chiroptic response and extinction spectra is observed. From 60 min to 120 min of reaction time, a marginal increase in chiroptic response and extinction occurred as morphology analysis showed a limited size increase while preserving generated chiral motif.

### Enantioselective interaction of nucleobase and gold high-index surface

**Fig. 3:**
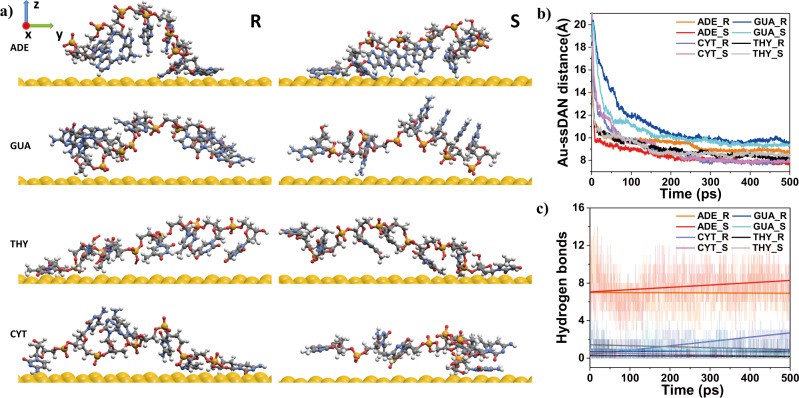
Molecular dynamics simulation of enantioselective interaction between gold surface and homoligomers. **a** Structure of ssDNA adsorbed on Au(321)^R^ (left) and Au(321)^S^(right) after 500 ps. From the top, Adenine, Guanine, Thymine, Cytosine. **b** Distance between the center of mass of Au surface and ssDNA. **c** The number of hydrogen bonds as a function of time.

The nucleobase-dependent chirality evolution can be correlated to the enantioselective interaction at the Au-ssDNA surface. The sequence-specific binding orientation at the interface is driven by the formation of chirality-dependent intra-strand hydrogen bonding. We performed an MD simulation to elucidate the potential interaction and structure of ssDNA on the Au surface. Based on the morphology analysis of ssDNA-induced chiral nanoparticle growth, high-index Au(321)^R/S^ and ssDNA oligomers were modeled for the MD simulation. Au(321)^R/S^ was chosen as a model high-index step-kink surface based on our previous understanding of intermediate high-index facets prior to chiral evolution^25,41^. For the simplicity and time efficiency of simulation, a 7-mer ssDNA oligomer of all types of homo-oligonucleotides was used throughout the simulations performed during a total of 500 ps. The spatial distance and orientation of chiral molecules on chiral surfaces can significantly affect the morphology during nanoparticle growth^42^. The relative orientations of ssDNA on Au surface are shown in Fig. 3a with the enlarged and aerial view of the interaction shown in Supplementary Fig. 7. The criteria for interaction was set to be less than 97% of the sum of van der Waals radii. It is based on the Au–H distance and has an average distance of 2.78 Å, which is approximately 97% of the Au–H with a van der Waals radii sum of 2.86 Å^43^. First, in the case of Adenine on the R surface, three bases interacted with gold surfaces through the –NH_2_ functional group, where the base at the 3′ end was attached almost parallel to the surface while the other bases were attached slightly away from the surface. On the contrary, the other four bases in the middle are clearly detached from the surface. In the case of adenine on the S surface, six out of seven bases are directly interacting with the surface. The base at the 3′ end of the oligomer lies flat with all atoms in close contact with the surface, while the subsequent three bases show an interaction through the –NH_2_ functional group, as shown in Supplementary Fig. 7. The remaining two bases interacted with the gold surface through the –NH_2_ functional group and carbon in a hexagonal ring, respectively. This result indicates that the Adenine oligomer interacts at the chiral surface with distinct enantioselectivity in terms of the number of binding bases and the surface orientation. On the contrary to the case of Adenine oligomer, no base in the Guanine oligomer made close contact with the base on the R surface, and only one base made contact with the S surface. In the case of Thymine oligomer, the orientation on the R and S surface was almost identical in which the base at the 3′ end made complete contact with the surface. However, two bases next to it were attached through the hydrogen of the –CH_3_ functional group. In the case of Cytosine oligomer, three bases contacted the R surface, while one base contacted the S surface. The Cytosine oligomer shows almost parallel contact with the surface in both R and S surfaces.

The temporal evolution of the perpendicular center-of-mass distance to the Au surface was calculated to quantitatively describe the enantioselective interaction between ssDNA and the Au surfaces. As shown in Fig. 3b, the distances between the center of the mass point of each nucleobase to the Au(321)^R/S^ surface were plotted with respect to time. Considering that all ssDNA showed the stabilized orientation and location of the molecules after 300 ps, the relative distance of each nucleobase from the surface was averaged for the last 200 ps. The spatial distance average of oligomer from the surface of the last 200 ps was found to be 8.87 Å (R) and 7.92 Å (S) for Adenine oligomer, 9.72 Å (R) and 9.42 Å (S) for Guanine oligomer, 8.36 Å (R) and 8.52 Å (S) for Thymine oligomer, and 7.86 Å (R) and 7.94 Å (S) for Cytosine oligomer. The center of mass distance for the Adenine oligomer shows a clear difference in the distance between R and S surfaces (0.95 Å). The differences in center of mass distance between R and S surfaces for Thymine, Guanine, and Cytosine oligomers are relatively small (0.30 Å, 0.16 Å, and 0.08 Å, respectively). Comprehensively, surface orientation and center of mass distance results indicate that Adenine oligomer on Au(321) surface shows the greatest enantioselective interaction, which supports the experimental data of Adenine oligomer selective chirality evolution.

The large enantioselectivity of Adenine-ssDNA can be understood from the formation of intra-strand hydrogen bonding. Surface interaction of single- or double-stranded DNA is directly correlated with the initial structure and stability of the oligomer which are closely related to their intra-strand interactions^44,45^. For example, intra-strand hydrogen bonds between the backbone carbonyl oxygen and neighboring nucleobases stabilize L-aTNA or SNA in helical structures^46^. Addition of Guanidinium-based drugs is known to promote intra-strand hydrogen bond formation to stabilize DNA structures to mitigate DNA mutation^47^. Furthermore, aromatic stacking of ssDNA is also known to influence oligomers’ conformation, stability, and interaction tendency^48–50^. The influence of intra-strand hydrogen bonding on oligomer structural stability and how it affects the surface interaction on the gold surface is shown in our simulation results. The nucleobases bind on the surface while maintaining specific orientations after 200 ps, indicating that intra-strand interactions also dictate the stabilized surface orientation. Therefore, it can be inferred that the selectivity of the R/S surface and the interaction between each base should be taken into consideration. For this reason, we closely examined the number of intra-strand hydrogen bonds of each nucleobase during the interaction with the chiral surfaces. In Fig. 3c, the number of hydrogen bonds over time was plotted for each nucleobase. The average number of hydrogen bonds for Adenine oligomers is found to be 7.5, while those of guanine, thymine, and cytosine are 0.75, 0.7, and 0.97, respectively. Adenine oligomer shows a significantly large number of hydrogen bond formations compared to other nucleobases. Especially in the case of the Adenine oligomer on the S surface, the hydrogen bond number with respect to time evolution shows a gradual increase, which indicates intra-strand hydrogen bond formation is favored as the Adenine oligomer stabilizes over the Au(321)^S^ surface. More specifically, the Adenine oligomer on either Au(321)^R/S^ surface had a similar number of hydrogen bonds at the initial stage of interaction. However, a significant increase in the number of hydrogen bonds in the case of Au(321)^S^ occurs as time increases. This trend in intra-strand hydrogen bond formation could be directly visualized in time-variant dynamics of change in Adenine oligomer surface orientation on Au(321)^R/S^, as shown in Supplementary Fig. 8. As time progresses, the base at the 3′ end is attached to the Au(321)^R/S^ surface, and the intra-strand hydrogen bonding network of Adenine oligomers on the R and S surfaces is broken. Adenine oligomer on Au(321)^R^ shows local adsorption to the surface with only three bases interacting at both terminals of each oligomer. However, on the Au(321)^S^, Adenine is sequentially attached to form a new intra-strand hydrogen bonding network with the increase in the number of hydrogen bonding. These results indicate that the specific enantioselective behavior of Adenine oligomers on Au(321)^R/S^ surfaces is closely correlated with the intra-strand hydrogen bond formation.

To confirm the accuracy of our simulation result, simulation time and oligomer length have been increased. In the 500 ps of the calculation, cytosine showed an increasing trend line of hydrogen bonds on the R surface. However, an increase in the simulation time to 2000 ps resulted in the overall constant trend of a number of hydrogen bonds, as shown in Supplementary Fig. 9. For the 2000 ps of simulation, Adenine on the S surface consistently showed an increasing trend in a number of the hydrogen bond. Furthermore, simulations were conducted with increased oligomer length to confirm the adequacy of the calculated results. As shown in Supplementary Fig. 10, an increase in oligomer length from 7-mer to 12-mer showed similar trends in the center of mass distance and the number of hydrogen bonds as a function of time. The average spatial distance difference between R and S surface for the last 200 ps was found to be 9.70 Å (R) and 9.20 Å (S) for Adenine, 10.41 Å (R) and 10.32 Å (S) for Guanine, 9.61 Å (R) and 9.73 Å (S) for Thymine, and 8.56 Å (R) and 8.75 Å (S) for Cytosine. While the overall center of mass distance increased as the length of the ssDNA increased, Adenine still showed significantly larger difference in the average center of mass distance with 0.5 Å. Furthermore, hydrogen bonding increased only in the case of Adenine on the S surface, which is again observable in the simulation result using 7-mer oligomers. These results infer that the calculation results remain consistent over the change in oligomer length to longer sequences.

DFT calculations were conducted to understand the adsorption energetics and enantioselectivity of Adenine oligomer. To understand the binding energy on each oligomer, mononucleobase forms were tested to find the adsorption orientation and relative binding affinity. The possible binding orientations of nucleobases were calculated based on the assumption of the presence of ssDNA backbone structure, while the kink site was considered the possible adsorption position based on the high adsorption affinity of kink in high-index planes as previously reported^51^. The adsorption energies for nucleobases on each possible binding position are shown in Supplementary Table 2 with the denotation of each position in Supplementary Fig. 11. The binding position determination process is explained using Adenine for convenience, and a similar determination process has been applied to other nucleobases as well. Adenine in DNA bonds with the pentose group through the N9 site. Therefore, despite five N atoms that can adsorb onto the kink, the N9 site and its adjacent N3 site were excluded from consideration due to backbone’s inherent geometry and position^52^. The remaining three sites were tested for potential adsorption on the kink site of the Au(321)^R^ to compare the adsorption energies. Twelve configurations were calculated for R surfaces by rotating the orientation by 30°. Finally, it was found that the N7 site showed the most stable binding site with –NH_2_ functional groups weakly binding to the surface. This result is consistent with the recently proposed mechanism of Adenine adsorption on the Au surface, where the significant interaction occurred via N7. They reported that the major interaction proceeds through the N7 site, and the –NH_2_ functional group also interacts weakly^33^. After finding the adsorption site of the nucleobases on R surfaces, the adsorption energetics were calculated in a similar manner on the S surface for further analysis.

**Fig. 4:**
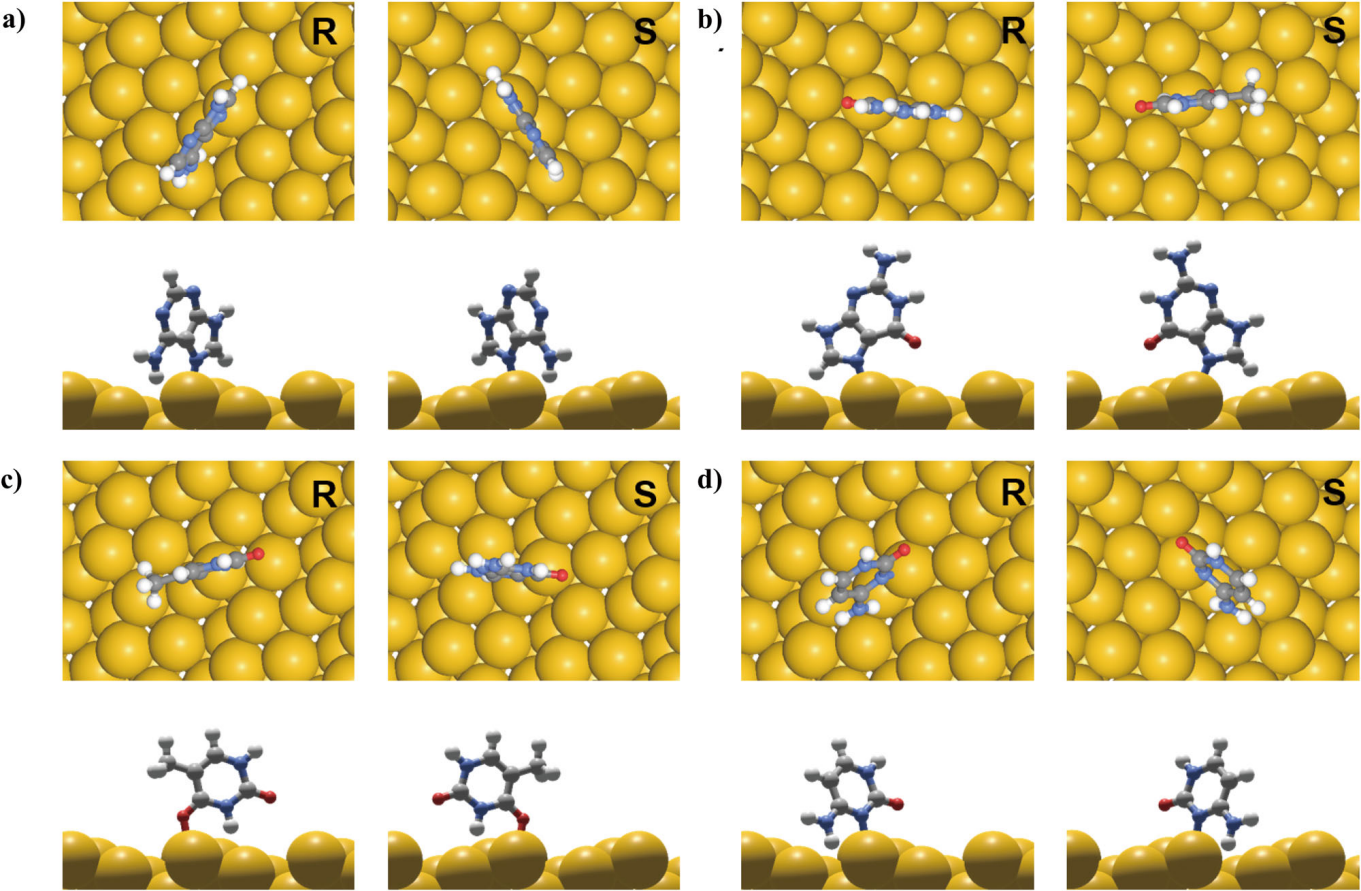
Binding orientation of nucleobases on Au (321)^R/S^ surfaces. The top and side view of DFT-optimized adsorption geometry of **a** Adenine, **b** Guanine, **c** Thymine, and **d** Cytosine on Au(321)^R^ (left) and Au(321)^S^ (right).

In Fig. 4a–d and Supplementary Table 2, the binding orientation of each nucleobase and their relative binding affinity on Au(321)^R/S^ are shown. The adsorption energy at the most stable site was found to be −1.465 eV (R surface) and −1.458 eV (S surface) for Adenine, −1.339 eV (R surface) and −1.335 eV (S surface) for Guanine, −0.482 eV (R surface) and −0.486 eV (S surface) for Thymine, and −1.586 eV (R surface) and −1.586 eV (S surface) for Cytosine. Based on this result, the enantioselectivity of each monomer was calculated by the difference of adsorption energies on R and S surfaces, which shows the values of 0.007 eV for Adenine, 0.005 eV for Guanine, 0.003 eV for Thymine, and 0.000 eV for Cytosine. Owing to the small scales of these values, it is possible to infer that the monomer form of nucleobases does not show significant enantioselectivity. This result agrees with our experimental result that the deoxyadenosine monophosphate monomer did not show a sign of chiral development, but Adenine oligomer showed chirality development with a sequence length dependency.

Insignificant enantioselective interaction of nucleobase at the surface allows the construction of a hypothesis that ssDNA induced chiral gold nanoparticle synthesis follows a different growth mechanism compared to the previous chiral gold nanoparticle synthesis using amino acids and peptides^25^. While previous chirality transfer occurs from chiral molecules themselves to the inorganic morphology, ssDNA induced chiral gold nanoparticle utilizes chirality transfer from oligomeric structures of composed nucleobases. In order to confirm this hypothesis, we extended the number of Adenine to represent the oligomer form of surface interactions more closely. The top and side view of optimized adsorption geometry of Adenine on each Au(321)^R/S^ by filling all the kink sites in the unit cell is shown in Supplementary Fig. 12 with a dashed line indicating intra-strand hydrogen bond formation. The adsorption energies of Adenine on R and S surfaces were calculated to be −19.726 eV (−1.644 eV/molecule) and −19.912 eV (−1.660 eV/molecule), respectively, with enantioselectivity to be 0.186 eV. This is a significant increase compared to that of the Adenine monomer, and even with normalization of enantioselectivity by the number of Adenine calculated, it still showed a significant increase to 0.016 eV per base. This increase could be attributed to the specific alignment of Adenine monomers on Au(321) surfaces, which promote intra-strand hydrogen bonding. From the top and side view, the Adenine monomers on the S surface show a relatively tilted orientation toward the gold surface than on the R surface and show a higher tendency to form intra-strand hydrogen bonds on the S surface than on the R surface. This result is in good agreement with our MD simulation results which show that the intra-strand hydrogen bonding network was a key factor in the enantioselectivity of oligomers by affecting their overall structures. Therefore, it suggests that the origin of chirality and surface enantioselectivity is related to the intra-strand hydrogen bonding formation of the Adenine oligomer.

### Sequence programmable chirality evolution

Oligomer sequence programming for selective modulation of chirality evolution could significantly extend the scope of nanomaterials synthesis using biomolecules. As the proof of concept, programming of oligomer composition and sequence pattern was conducted to control the binding orientation of oligomers at chiral surfaces and modulate the chirality evolution. Simulation results indicate that the enantioselective interaction at the nanoparticle surface is closely related to the oligomer adsorption orientation, which depends on oligomer length and nucleobase types. This is a different mechanism of biomolecules-induced chirality evolution, as previous synthesis methods utilized molecular chirality itself rather than their oligomeric surface geometry and compositions. Therefore, for the efficient control of various sequence patterns within the same oligomer length, 12-mer Adenine-based oligomers were chosen with cytosine as sequence pattern modulator. All growth conditions remained constant for each nanoparticle from the method section, while the ssDNA oligomer was replaced with the designed oligomer sequences. The effect of sequence modulation was analyzed by changes in CD response and SEM-based morphology.

**Fig. 5:**
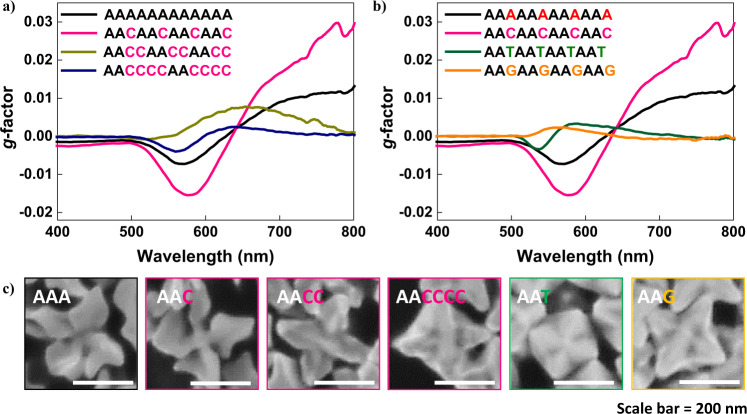
Oligomer sequence-programmed chirality evolution. **a** The chiroptic response of chiral gold nanoparticles synthesized with a 12-mer oligomer with consecutive Cytosine spacer length control from 0, 1, 2, and 4. **b** The chiroptic response of chiral gold nanoparticles synthesized with a 12-mer oligomer with spacer nucleobase control while maintaining consecutive Adenine length constant at 2. **c** High-magnification SEM image of sequence-programmed gold nanoparticle synthesis.

Figure 5 shows the chiral development of ssDNA-induced chiral gold nanoparticles with respect to the oligomer sequence pattern. Figure 5a, b shows the chiroptic response of nanoparticles synthesized using sequence patterned oligomers. While the consecutive Adenine nucleobase number remained constant within the total length of 12-mer, the number of cytosine spacers was gradually increased to induce change in chirality development. In previous experiments, only oligonucleotides composed of Adenine nucleobase showed chirality evolution property during nanoparticle synthesis. However, as shown in Fig. 5a, the addition of one Cytosine spacer after two consecutive Adenine nucleobases induced a higher maximum chiroptic response. The chiroptic response of Adenine 12-mer showed a negative g-factor of 0.007 at 570 nm and a positive plateau g-factor of 0.01 at above 700 nm wavelength. However, AAC patterned oligomer showed an enhanced g-factor with a negative peak of 0.015 at 579 nm and a positive peak of 0.03 at near 800 nm. On the contrary, further increase of Cytosine spacer numbers to 2 and 4 showed a gradual decrease of maximum chiroptic response. Furthermore, while the Cytosine spacer number was maintained constant, the number of consecutive Adenine number has been altered to test its effect on chiral development as shown in Supplementary Fig. 13. While AAC patterned oligomer sequence remained to show the highest chiroptic response, variation of total or consecutive Adenine number within the oligomer showed a dynamic change in chiroptic response in terms of peak positions, peak intensities, and spectral shapes. This result clearly demonstrates that sequence patterns critically affect current synthetic systems’ chiral development.

The spacer nucleobase type also critically affects the chiroptic response generated, as shown in Fig. 5b. While the sequence pattern was kept constant to two consecutive Adenine and one spacer, the spacer nucleobase was varied. With the spacer change to Thymine and Guanine, the chiroptic response decreased intensity to a max g-factor of below 0.005 in both negative and positive CD. Furthermore, Thymine and Guanine spacers show a blue shift of both negative and positive peaks compared to no spacer or Cytosine spacer. These chiroptic response changes using spacer length and spacer type could be more directly visualized using morphology analysis shown in Fig. 5c. Prior to introducing spacer, A12 oligomer-induced chiral nanoparticles showed distinct chiral arm structures that slightly overlap each other. However, introducing one Cytosine spacer increased the maximum chiroptic response by generating a gap between each chiral feature. Gap structures between chiral features are known from our previous simulation results to induce electric and magnetic field confinement, which directly affect the magnitude of chiroptic response^25,53^. Further increase of spacer length or change in spacer nucleobase type induced different gap morphology between <111> protruded arm structures and different facet generations, which change resulting chiroptic response.

Based on our current results, sequence patterns along with the energetic favorability of nucleobases at the surface comprehensively affect the final nanoparticle morphology and their chiroptic response. Such sequence-dependent versatility in chiral nanomaterials may further expand the potential applicability. Nanoparticle morphology and their chirality control induce change in their cellular dynamics with potential controllability using polarized light in targeted delivery or therapy^54–56^. Nucleobases hold unique binding tendencies with different metal surfaces, so a change of materials selection from gold to other noble metals such as silver or copper could diversify synthesized chiral nanomaterials. For example, bimetallic nanoparticle morphology control using ssDNA oligomers previously showed different structural evolution tendencies compared to that of monometallic nanoparticle synthesis^36,57^. While expansion of materials selection or test of biomedical applicability is beyond the scope of current work, we envision that designing oligomers or use of short functional ssDNA could expand to the realization of programmable chiral nanomaterials for biomedical applications.

In conclusion, single-nanoparticle-level chiral nanoparticles were synthesized using Adenine-based ssDNA. While other nucleobases showed no chirality evolution capability, Adenine-based oligomers showed a distinct chirality evolution. Furthermore, chirality generation showed a variation in a dominant sign of chiral response with respect to change in oligomer length and relative CTAB concentration with dissymmetric factor reaching g ~ 0.04 at 650 nm. The A50 oligomer-induced chiral gold nanoparticle showed a generation of 4 chiral arms with a counter-clockwise rotation of the chiral motif. Each chiral arm structure protrudes outwards from the center point in each plane to show octopod-like outer boundaries. The simulation study revealed that each nucleobase on the gold surface shows chirality-dependent geometric orientation and adsorption energy differences. Adenine showed the highest enantioselectivity with the most distinguishable surface orientation difference on R and S surfaces. Furthermore, this change in orientation was also observable in adsorption energy difference which is 0.016 eV/molecule (i.e. 1.544 kJ/mol) for Adenine between R and S surfaces. This significant enantioselective molecular orientation or binding energy difference coincided with the Adenine-specific chirality evolution capability. Utilizing this nucleobase specific interaction, Adenine-based oligomer sequence was programmed to modulate chirality development. Depending on spacer length, consecutive Adenine length, and the type of the spacer, each chiral nanoparticle showed a difference in the development of chiral features and resulting chiroptic response. While the exact mechanism of gold-ssDNA interaction to induce chirality is yet to be fully understood, we believe that our experimental and simulation results provide a starting point for chiral nanomaterials synthesis with programmable optical and geometrical properties.

## Methods

### Materials

All chemicals and oligomers were used as received without any modification or further purification. Hexadecyltrimethylammonium bromide (CTAB, 99%), Cetyltrimethylammonium chloride solution (CTAC, 25 wt% in H_2_O), Potassium Iodide (KI, 99.5%), L-ascorbic acid (AA, 99%) and tetrachloroauric(III) trihydrate (HAuCl_4_·3H_2_O, 99.9%) were purchased from Sigma-Aldrich. All single-stranded oligonucleotides were synthesized and purchased from Integrated DNA Technologies, Inc. (IDT). All aqueous solutions were prepared using high-purity deionized water (18.2 MΩ cm^−1^).

### Synthesis of octahedron seed nanoparticles

The gold octahedron seed nanoparticle synthesis was conducted based on the previously reported method^38^. At first, small 2-nm-sized spherical nanoparticles were synthesized using a 320 mg of CTAC in 9.5 mL of deionized water, 0.25 mL of 10 mM HAuCl_4_, and 0.45 mL of 20 mM NaBH_4_. The following solution was incubated for 3 h to decompose the remaining NaBH_4_. Then, the 1/10 diluted spherical seeds were grown in the growth solution containing 9.5 mL of deionized water, 0.2 mL of 10 mM HAuCl_4_, 320 mg of CTAC, 5 μL of 10 mM KI, and 0.22 mL of 40 mM AA. After 15 min of incubation, the reaction was stopped and the resultant crystals were washed twice by centrifugation (6708 × *g*, 150 s) and redispersed in a 1 mM CTAB solution.

### Synthesis of ssDNA induced chiral gold nanoparticles

In the typical synthesis of ssDNA-induced chiral gold nanoparticle, 0.8 mL of 150 mM CTAB and 0.100 mL of 10 mM gold chloride trihydrate were introduced to 3.95 ml of deionized water for the formation of an [AuBr_4_]^−^ complex. Upon the addition of 0.475 mL of 100 mM l-ascorbic acid solution as the reducing agent, Au^3+^ reduced to Au^1+^. As the chirality-inducing agent, 25 μL oligonucleotide was introduced to meet the desired final concentration. Finally, for the initiation of nanoparticle growth, 25 μL prepared octahedron seed dispersed in 1 mM CTAB solution is introduced. For the in situ nanoparticle synthesis, 3 mL of the above-mentioned nanoparticle growth solution was transferred into a 1 cm × 1 cm quartz cuvette and time-variant optical response measurement was conducted for 2 h with 5-min intervals.

### Optical and morphological analysis

For the optical analysis, extinction and circular dichroism (CD) spectra were measured through a J-815 spectropolarimeter instrument (JASCO). Based on the acquired data from the spectropolarimeter, Kuhn’s dis-symmetry factor, g-factor, was calculated for the quantitative comparisons of chiroptic responses among different nanoparticles. For morphology analysis, Scanning Electron Microscopy (SEM) images were taken with a SUPRA system (Zeiss). For the angle measurement of chiral arm structures, SEM images of nanoparticles have been analyzed using Image J program. Furthermore, the modeling of the nanoparticle morphology has been conducted on McNeel Rhinoceros 3D 5.0, based on analysis of SEM images taken from various angles.

### Simulation of enantioselective interaction at Au-ssDNA interface

#### MD method

All molecular dynamics (MD) simulations were conducted by using the large-scale atomic/molecular massively parallel simulator (LAMMPS) package^58^. For ssDNA and Au, CHARMM-METAL force field was used^59^. For the water model, the TIP3P model was used^60^. The simulations used particle mesh Ewald (PME) method and temperature of 298.15 K in the NPT ensemble. A 4-layered (9 × 18) surface unit cell of Au(321) was used, and the length of ssDNA was 7-mer. To mimic the ionic conditions, NaCl salts were added. The simulation box size was 71 Å × 112 Å × 75 Å, which was large enough to ensure that there are no interactions between ssDNA through the periodic boundary condition. Data analysis was conducted by VMD^61^. Hydrogen bond was calculated by cutoff length of 3.3 Å, and cutoff angle 90°.

#### DFT method

Density functional theory (DFT) calculations were performed using the Vienna Ab initio simulation package (VASP) to find the adsorption geometry of the base part of ssDNA on Au(321) surface^62^. The Perdew–Burke–Ernzerhof (PBE) generalized gradient functional along with the projector augmented wave (PAW) method was employed to describe ionic core^63^. DFT-D3 was employed to take into account the van der Waals interaction. A plane wave expansion with a cutoff of 400 eV was used with 2 × 2 × 1 Monkhorst-Pack *k*-point sampling of the Brillouin zone for the total energy calculations. The calculations were conducted using the residual minimization method for electronic relaxation, and accelerated using Methfessel-Paxton Fermi-level smearing with a width of 0.1 eV. For nucleobase adsorption calculation on Au surface, the existence of backbone morphologies was assumed. A 4-layered (3 × 3) surface unit cell with a vacuum spacing of 15 Å was used, on which the molecule was placed in different initial configurations by 30°. Total 12 initial configurations were considered for each nucleotide on R and S surfaces.

The adsorption energy, *E*_ads_, is defined as below.

1 $${E}_{{{{{{\rm{ads}}}}}}}=({E}_{{{{{{\rm{total}}}}}}}-{E}_{{{{{{\rm{slab}}}}}}}-{E}_{{{{{{\rm{base}}}}}}}\times {n})/{n},$$

where *E*_total_ is the total energy of system, *E*_slab_ is the energy of bare slab surface, *E*_base_ is the energy of a base molecule in gas phase, and *n* is the number of adsorbates. With this definition, negative adsorption energy corresponds to the energetically favored adsorption on the surface. To characterize the enantioselectivity of adsorption, we used the difference in adsorption energies and compared using the absolute value.

## Supplementary information

Supplementary Information

## Data Availability

The authors declare that the main data supporting the findings of this study are available within the article and its Supplementary Information files. Furthermore optical and structural analysis is provided in the source data file.
