# Supplementary material for: Adenine oligomer directed synthesis of chiral gold nanoparticles
Source: Nat Commun. 2022 Jul 2;13:3831. doi: 10.1038/s41467-022-31513-y (PMC9250518; doi:10.1038/s41467-022-31513-y)
Supplement: Supplementary file 1 — Supplementary Information [file 41467_2022_31513_MOESM1_ESM.pdf]

Supplementary Information

Adenine Oligomer Directed Synthesis of Chiral Gold Nanoparticles

Cho et al.

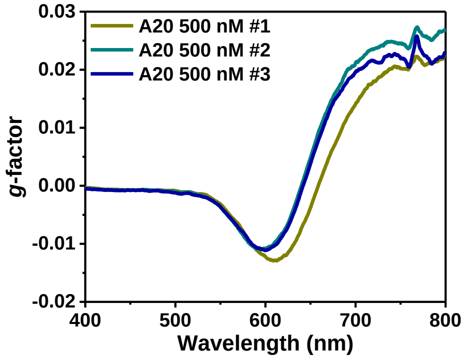

Supplementary Figure 1. Repetitive synthesis of A20 oligomer at 500 nM

Three repetitive synthesis of A20 oligomer at 500 nM concentrations. Under the exactly same synthesis condition, experiments were conducted on three different dates to check the reproducibility.

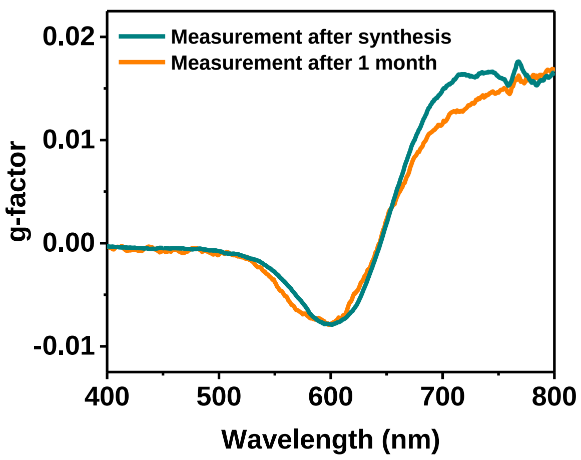

Supplementary Figure 2. Chiroptic response stability of Adenine induced chiral gold nanoparticle.

Synthesized nanoparticles were initially analyzed then stored in 1 mM CTAB solution at room temperature for 1 month before the 2nd measurement.

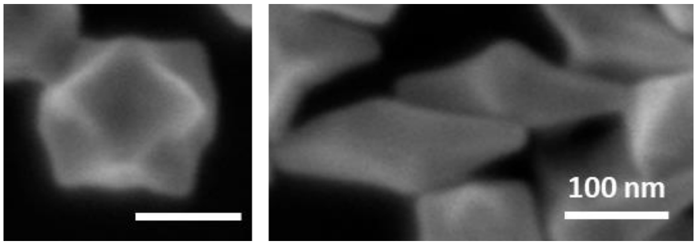

Supplementary Figure 3. Morphology of nanoparticles synthesized using deoxyadenosine monophosphate monomers (dAMP)

Enlarged SEM images of nanoparticles synthesized using dAMP. Nanoparticles were mainly composed of high-indexed trisoctahedral nanoparticles and bipyramid morphologies.

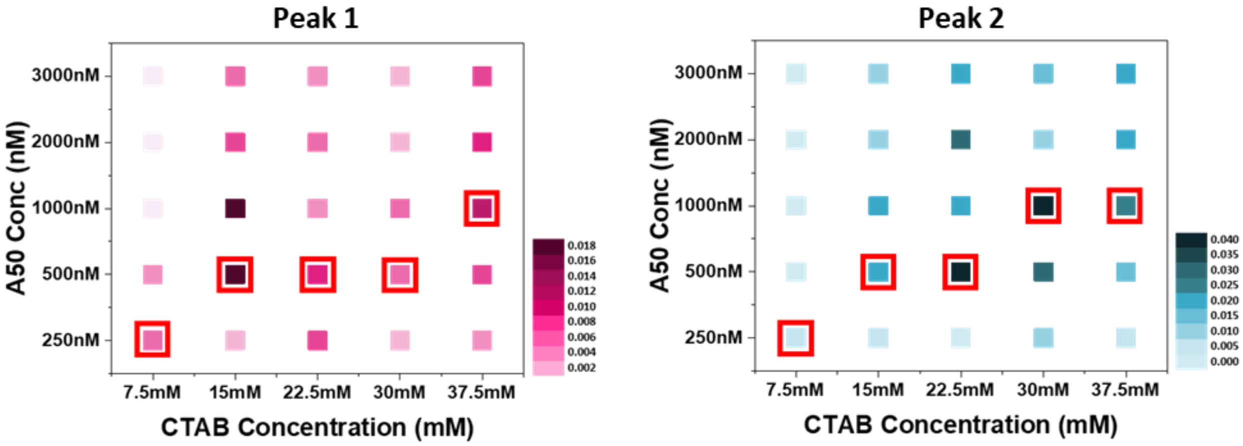

Supplementary Figure 4. *g*-factor diagram of A50 oligomer and CTAB concentration variation

Maximum *g*-factor of synthesized chiral gold nanoparticle using variation of relative CTAB and A50 oligomer concentrations. Max *g*-factors have been recorded regardless of peak positions. Peak 1 indicates the recording of the negative sign maximum *g*-factor peak and peak 2 indicates the recording of the positive sign maximum *g*-factor peak.

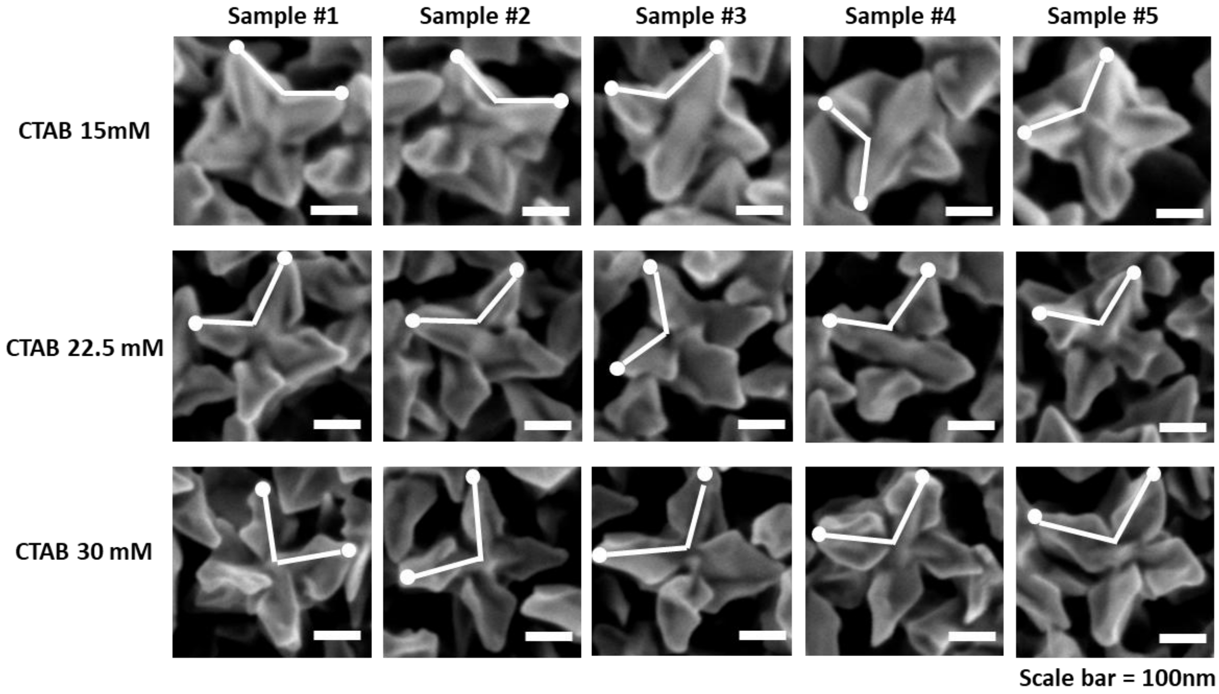

Supplementary Figure 5. SEM images of A50 induced chiral gold nanoparticle with CTAB concentration variation

SEM morphology analysis of CTAB variation in A50 oligomer induced chiral gold nanoparticles. All synthesis conditions have remained constant except of the CTAB concentrations.

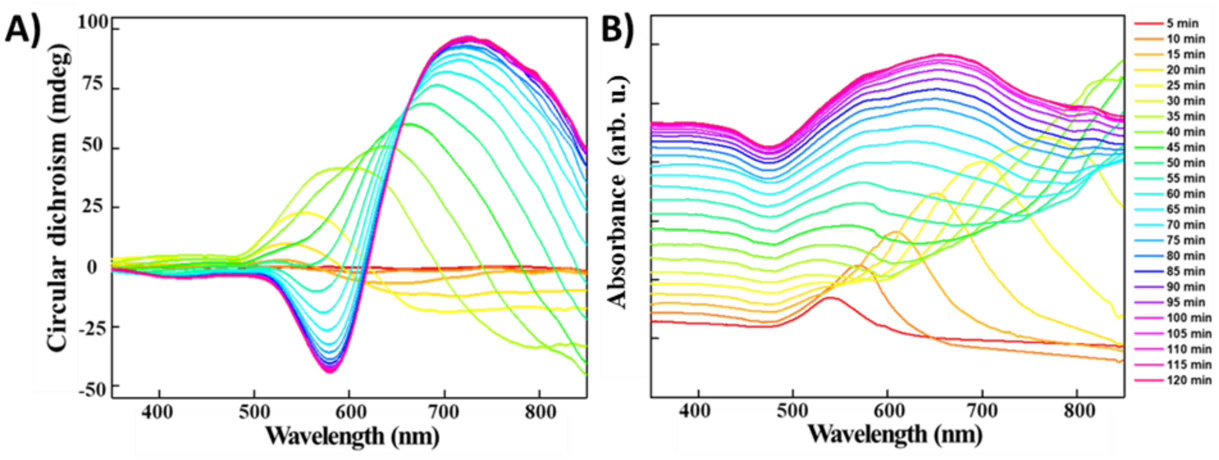

Supplementary Figure 6. Time-variant CD and extinction graphs of DNA induced chiral gold NP

Time-variant A) CD and B) Extinction measurement data. Data was measured in a 5 minute intervals while reaction proceeded inside the quartz cuvette.

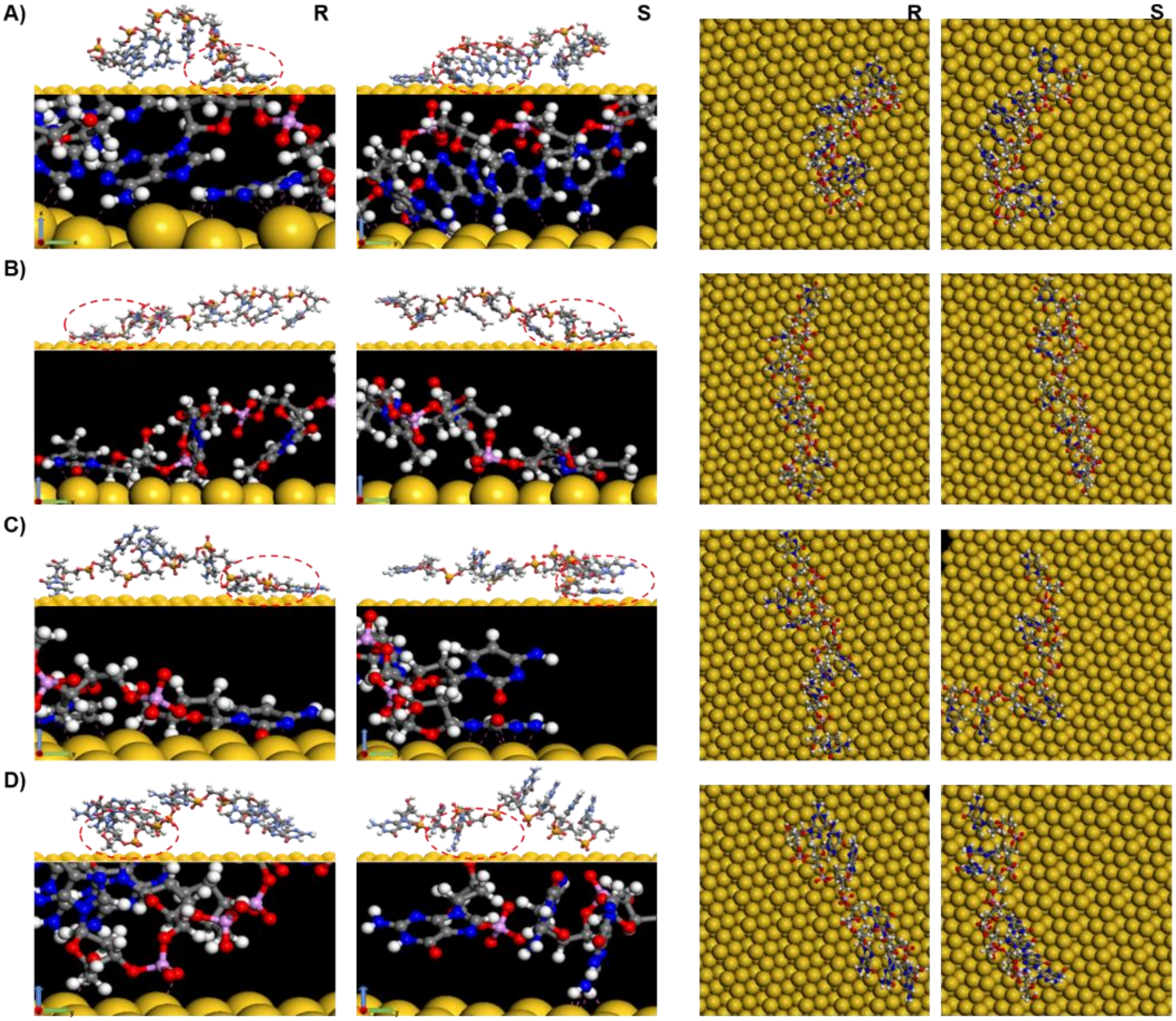

Supplementary Figure 7. Enlarged and aerial MD simulation view of Au-nucleobase interaction

Enlarged and aerial view of the bases interacting with the Au surface. The side view and top view of MD simulations are in the order of A) adenine, B) thymine, C) cytosine, D) guanine. The part marked with a red dash line circle is the part viewed more closely. Furthermore, surface-molecule interactions are expressed with a purple dash line in the side view.

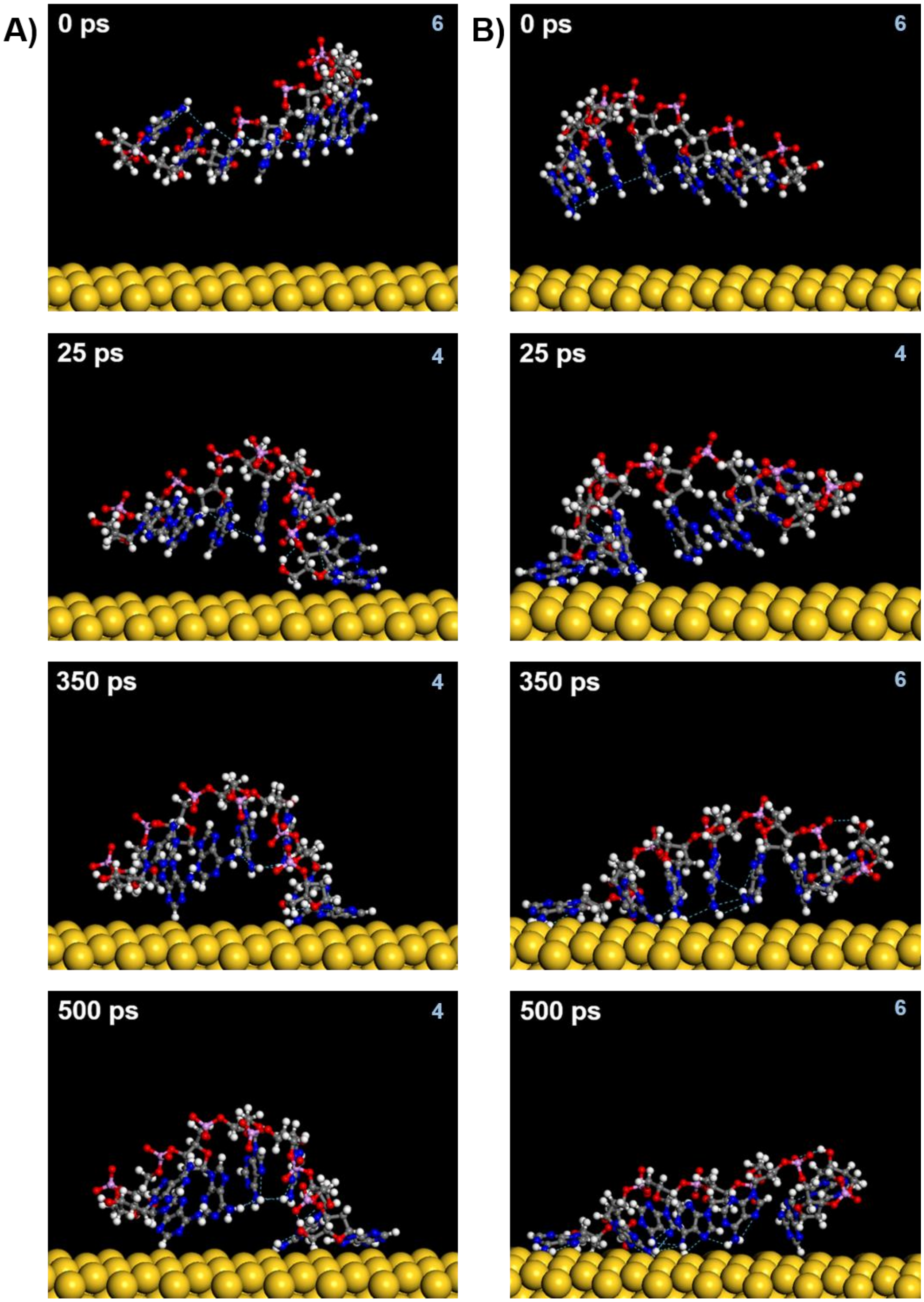

Supplementary Figure 8.Time-variant dynamics of Adenine oligomer on Au(321)R/S surface

Time-variant adenine orientation on A) $(321)^R$ surface, B) $(321)^S$ surface. Blue dash line represents hydrogen bonds and the right top number is the number of inter-base hydrogen bonds.

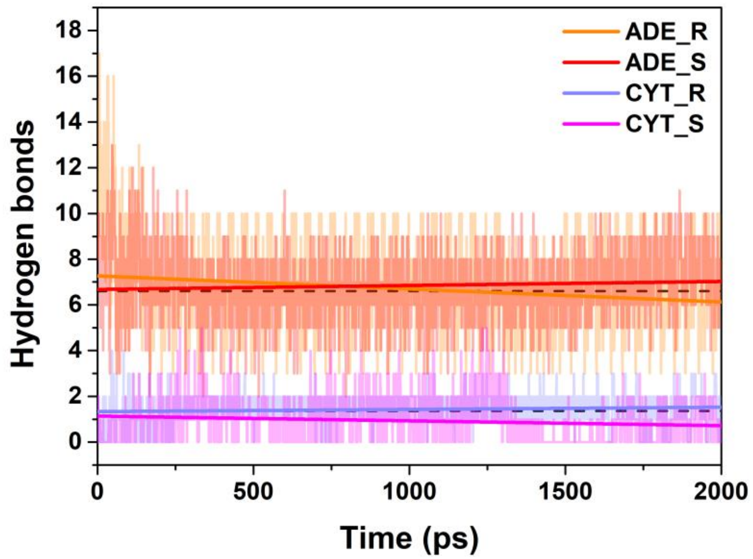

Supplementary Figure 9. Change in hydrogen bond of Cytosine and Adenine for extended simulation time.

The simulation of change in hydrogen bond number with 4 times longer calculation time. While Adenine displays consistent increase in number of hydrogen bonds, cytosine shows overall decrease of number of hydrogen bonds. Dashed black lines are reference parallel line to indicate change in number of hydrogen bonds.

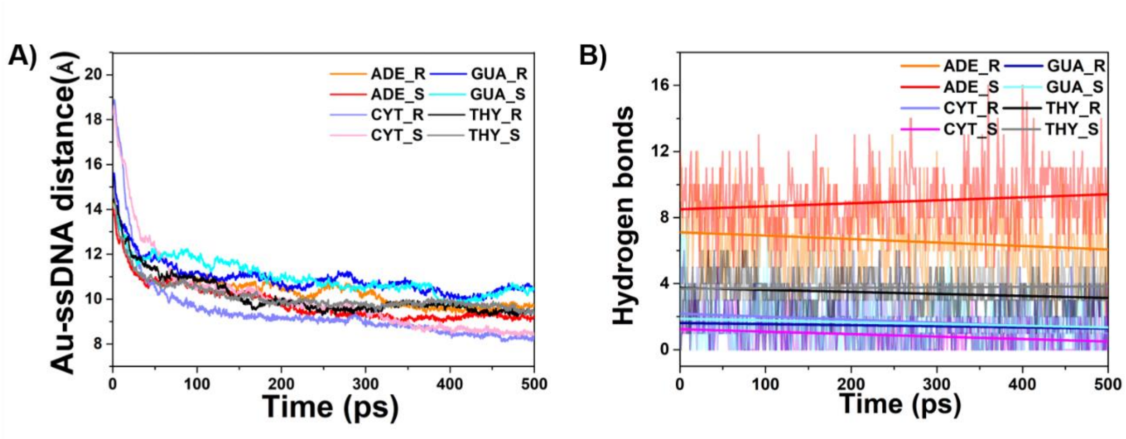

Supplementary Figure 10. Molecular dynamics simulation of enantioselective interaction between gold surface and homoligomers with increased oligomer length. A) Structure of ssDNA adsorbed on Au(321)^R (left) and Au(321)^S(right) after 500ps. From the top, Adenine, Guanine, Thymine, Cytosine. B) Distance between the center of mass of Au surface and ssDNA. C) The number of hydrogen bonds as a function of time.

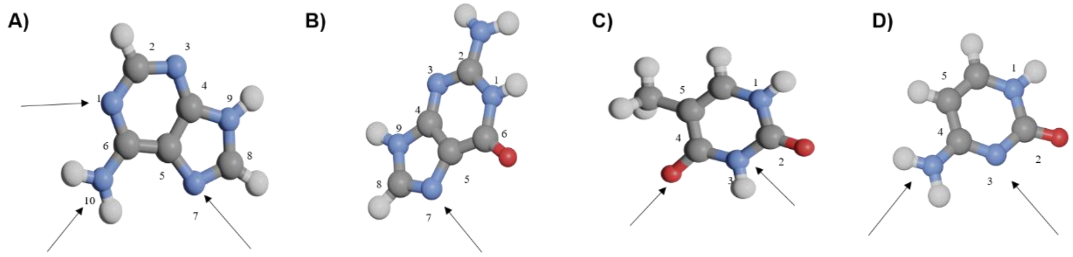

Supplementary Figure 11. Possible adsorption sites of four bases.

Considering the backbone structure, the sites indicated by the arrow are possible surface interaction sites of A) adenine, B) guanine, C) thymine, and D) cytosine.

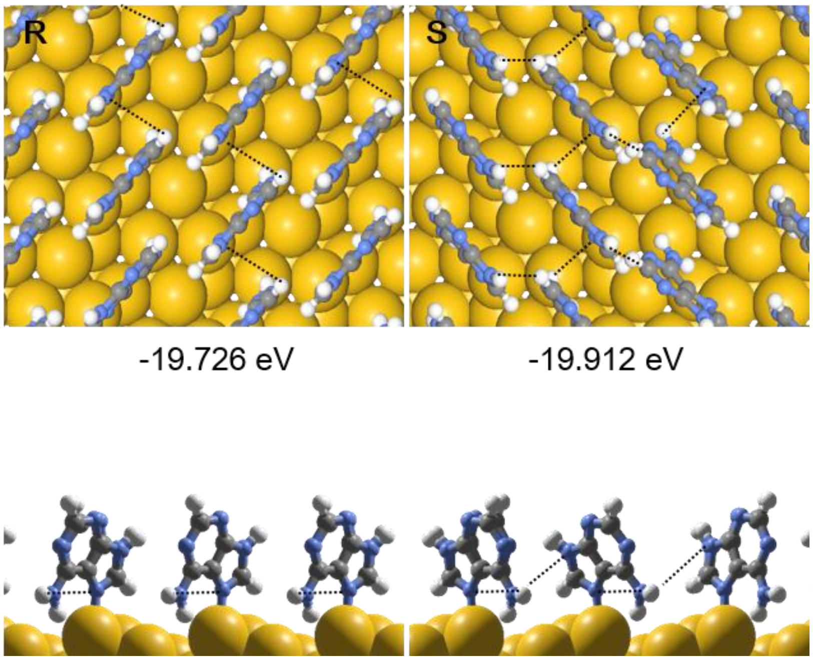

Supplementary Figure 12. DFT-optimized adsorption geometry of adenine on Au(321)$^{R/S}$
The top and side view of adenine on Au(321)^{R} (left) and Au(321)^{S} (right). The hydrogen bonds are expressed by dash line.

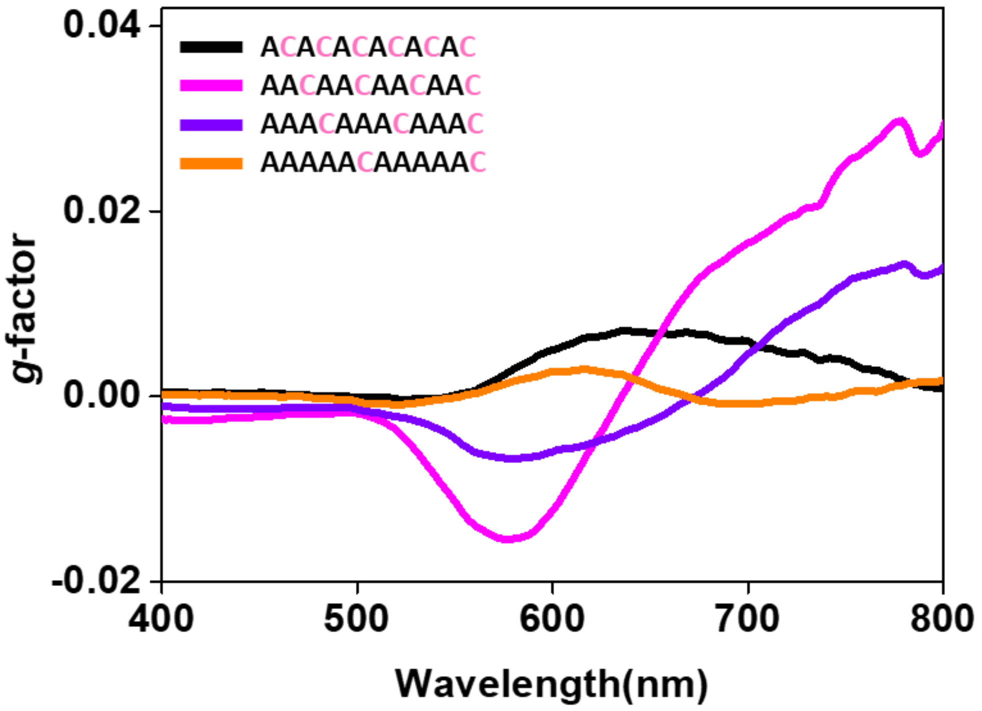

Supplementary Figure 13. Chiroptic response changes while varying Adenine sequence length and constant Cytosine spacer

Change in chiroptic response respect to change in Adenine sequence length while maintaining the spacer Cytosine length constant

	Sample # 1	Sample # 2	Sample # 3	Sample # 4	Sample # 5	Average
**CTAB 15 mM**	130.74	121.74	130.35	129.48	126.52	130.82 (±3.75)
**CTAB 22.5 mM**	109.28	118.25	114.61	116.43	120.25	115.76 (±4.19)
**CTAB 30 mM**	89.43	105.83	107.61	106.23	98.51	101.52 (±7.63)

Supplementary Table. 1. Angle measurement of chiral gap structures under CTAB concentration controlled synthesis environment.

Each synthesis conditions were maintained equal while only the input CTAB concentrations were varied as indicated. Total of 5 single nanoparticles for each synthetic conditions were measured.

	Adenine		Thymine		Cytosine		Guanine
**N1**	-1.40	**N3**	-	**N3**	-1.59	**N7**	-1.34
**N7**	-1.46	**O8**	0.48	**N7**	-0.02		
**N10**	-0.62						

Supplementary Table. 2. The adsorption energy of possible sites on Au(321)^{R}.

Most stable adsorption energy of possible sites at each base shown in Fig S8. The system adsorbed by the Thymine N3 site was unstable and thus the calculation was not performed.
